# On Neuropsychiatric Manifestations of Basal Ganglia Injury: A Report of Three Cases and Literature Review

**DOI:** 10.1155/2019/3298791

**Published:** 2019-04-04

**Authors:** Heela Azizi, Alexander Kilpatrick, Olaniyi Olayinka, Olusegun Poopola, Maleeha Ahmad, Alexa Kahn, Tasmia Khan, Dina Rimawi, Shantale Williams, Sinthuja Jayaraj, Ivan Leung, Sherina Langdon, Mirna Iskander, Ali Chohan, Deepa Nuthalapati, Ulunma Umesi, Geetha Vyas, Ayotomide Oyelakin, Kodjovi Kodjo, Chiedozie Ojimba, Oluwole Jegede, Carolina Nisenoff, Ayodeji Jolayemi

**Affiliations:** ^1^American University of Antigua College of Medicine, Department of Psychiatry, Interfaith Medical Center, Brooklyn, New York, USA; ^2^Department of Psychiatry, Interfaith Medical Center, Brooklyn, New York, USA; ^3^Medical University of the Americas, Department of Psychiatry, Interfaith Medical Center, Brooklyn, New York, USA; ^4^Saba University School of Medicine, Department of Psychiatry, Interfaith Medical Center, Brooklyn, New York, USA

## Abstract

The basal ganglia have been considered to primarily play a role in motor processing. A growing body of theoretical and clinical evidence shows that in addition to the motor functions the basal ganglia play a key role in perceptual and visual disturbances. This role may be evident in patients with basal ganglia pathology and subsequent manifestation of symptoms that include cognitive, perceptual, and affective disturbances. We present three cases with basal ganglia pathology that demonstrate affective and psychotic symptoms. Two of the cases presented with late onset psychotic disturbances suggesting likely neurological etiologies. The third case presented with treatment refractory psychosis and symptoms that are rare for a diagnosis of schizophrenia. The role of incidental bilateral basal ganglia calcifications in all the cases is discussed. A review of current literature highlighting various neuropsychiatric manifestations of basal ganglia pathologies in various patients with psychiatric symptoms is presented.

## 1. Introduction

The basal ganglia neural circuits are utilized in movement selection, planning, and learning. A much less researched feature of the basal ganglia is its function in cognition and perceptual function [1–3]. A few cases in literature have suggested the possible link between basal ganglia damage and visual hallucinations and psychosis [4, 5]. The symptoms exhibited by patients are consistent with varying degrees of hallucinations, delusions and flashbacks [6, 7]. The utilization of advanced imaging techniques such as Magnetic Resonance Imaging (MRI) might provide the means to identify organic causes of new onset psychoses and further knowledge of the functions of the basal ganglia. These lesions and associated symptoms give us insight into the complex pathways within the basal ganglia that have not been fully explored. A comprehensive investigation exploring damaged basal ganglia will allow us to elucidate potential causes of psychosis and hallucinations and open up new avenues of pharmacotherapy targeting the neural networks involved in hallucinations and psychosis. We present three patients with affective and psychotic symptoms who share brain imaging findings of incidental basal ganglia pathology. We also review relevant literature of symptoms reported for patients with bilateral basal ganglia calcifications (BGC).

## 2. Case Presentations

### 2.1. Case One

The first case was a 59-year-old African American male with a past medical history notable for schizoaffective disorder, depression, and substance abuse who was brought in to the emergency room for disorganized behavior and agitation in the community. At the time of admission the patient demonstrated disorientation, repetitive motor behavior, and an alternation between agitation and psychomotor retardation. He had poor response to communication and tactile stimuli. A suspicion of altered mental status due to organic causes was suspected with the possibility of catatonic excitement and retardation. He was admitted to the medical floor, with a work-up revealing a positive toxicology screen for cocaine and opioids. The patients CBC and BMP were within normal limits except for his ammonia level which was 80 mg/dl. The patient was initially treated with Chlorpromazine Hcl 50 mg orally daily for his agitated behavior as well as Naltrexone 50 mg orally daily for his opiate intoxication.

The patient exhibited incoherent thought process in addition to mumbled speech that made a significant portion of his assessment evaluation difficult. During evaluation, he displayed abnormal movements of his arms and face, with tremors and restlessness. His affect was flat. He did not display any perceptual disturbances or delusions. An assessment for cognitive impairment was noncontributory during his most recent admission. The patient received Mirtazapine 45 mg orally at bedtime and Olanzapine 10 mg orally daily in his treatment and by day three of admission had shown improvement in his disorganized behavior with supportive care. The patient demonstrated more effort to directly communicate with house staff after treatment began.

The patient reported a past history of psychiatric illness that was late in onset. His first presentation at the age of 51 years was significant for depressed mood, paranoid delusions, and auditory hallucinations for which he was diagnosed with a major mood disorder. His symptoms responded poorly to medications including antidepressants. His disease course involved increasing periods of impulsive behavior and agitation. He became noncompliant with his prescribed medications. He was later admitted to the medical floors at the age of 54 years for “repetitive behavior” during which he was found moving from his bed to the bathroom repeatedly as if he wanted to use the bathroom all the time. He also showed some abnormal rocking movements during this time period. A medical work-up for seizure was negative. He was discharged with a presumptive diagnosis of a psychotic disorder. Thereafter, at the age of 56 years he had an episode of property destruction in the community and it was noted that he had “abnormal body movements” in addition to lability of mood. His diagnosis was revised to schizoaffective disorder and he was treated for mood lability at the time with risperidone.

**Figure 1:**
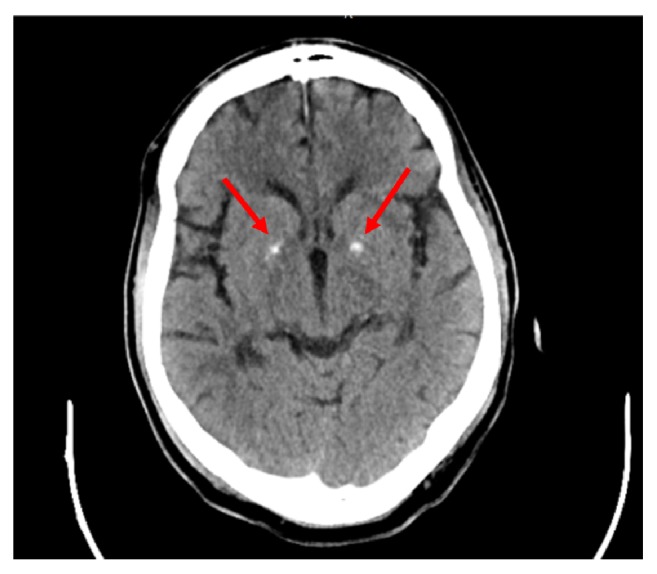
CT scan without contrast showing bilateral basal ganglia calcification.

Given the late onset of his neuropsychiatric symptoms, a computed tomography scan (CT) of his brain was done during his presentation, as seen in Figure 1. Reviewing his chart, it was noted that the calcifications were apparent in his first head CT taken in January of 2012 with no changes to the current CT in January of 2019.

### 2.2. Case Two

**Figure 2:**
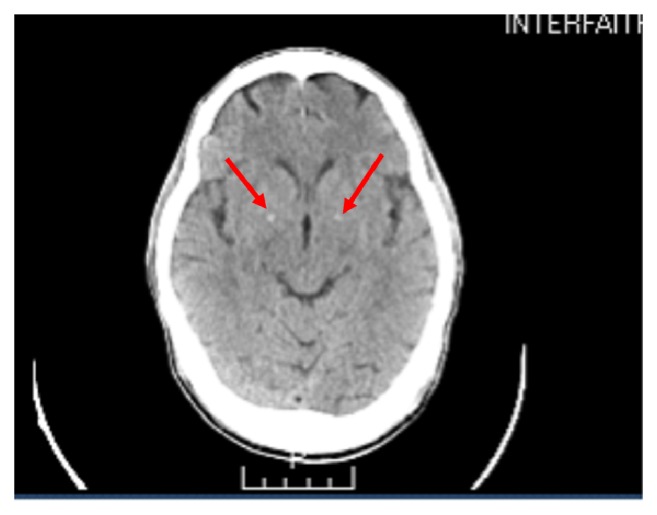
CT scan without contrast showing bilateral basal ganglia calcification.

The second case is of a 71-year-old African American man, who presented following an episode of agitation and combativeness in the community. The patient had a late onset psychiatric history starting at the age of 57 with a diagnosis of schizophrenia. He was found to demonstrate symptoms of pressured speech, loose association, paranoid delusions, grandiose delusions, and lability of mood. He was very restless during the interview and paced around multiple times as he could not sit still. He also demonstrated constant oral movements initially thought to be responding to internal stimuli. However, he denied auditory hallucinations and stated he could not control his mouth movements. A mental status examination (Montreal Cognitive Assessment) revealed a score of 22/30 with significant deficits in memory and executive control. This was consistent with his psychiatric evaluation which revealed significant confabulation in his history. The patient was started on haloperidol 2 mg orally twice daily and risperidone 0.5mg orally twice daily for his psychotic symptoms. The patient reports he has been noncompliant with medications previously. On systemic review, the patients CBC was within normal limits and he had a negative toxicology screen. The patients BMP had abnormal glucose values of 172 mg/dl and vitamin D level of 6.4ng/dl. An initial CT scan at the time of first diagnosis noted basal ganglia calcifications. A repeat CT scan of the brain was done (shown in Figure 2) and showed small bilateral basal ganglia calcifications, consistent with the image findings during his first episode of psychotic disturbance.

The patient showed some improvement in lability with medication compliance but his cognitive impairment, paranoid delusions, and grandiose delusions did not significantly improve. His extrapyramidal symptoms were not amenable to pharmacological interventions of anticholinergic benztropine.

### 2.3. Case Three

**Figure 3:**
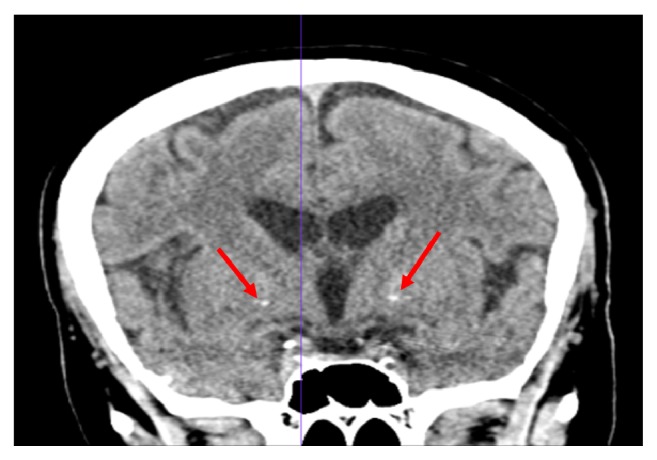
CT scan without contrast showing mild to moderate bilateral periventricular and deep white matter low attenuation along with trace bilateral basal ganglia calcification.

The third case reported is a 69-year-old English/Creole speaking Haitian female. Her initial admission was for an acute episode of mixed mood symptoms and psychotic symptoms at the age of 61. The patient reported constant restlessness with inner anxiety and preoccupation with delusions of control. She had a past history of treatment for chronic progressive paranoid delusions, cognitive dysfunction, and disorganized thought believed to be due to schizophrenia that responded poorly to treatment. At the time of her initial admission to our clinic, the diagnosis was revised to schizoaffective disorder considering the mood disturbances. A change in medications from risperidone 3 mg orally twice daily to fluphenazine 5 mg orally twice daily was also done. She showed no improvement in her psychosis and affective symptoms. At the time of her second admission, the patient was brought in by husband on account of bizarre behavior and disorganized thought in the context of medication noncompliance. Her symptoms had evolved to include visual hallucinations of Buddha, visual hallucinations of demons, and perceptual distortions of the floor. She endorses bizarre delusions stating there is a demon inside of her and that an “agent” took the place of her husband. She also exhibited depressive symptoms with worsening restlessness and cognitive functioning. Urine toxicology was negative on admission, with full blood count and metabolic panel within normal limits. Risperdal 2 mg orally twice daily was continued for psychosis. Paliperidone 156 mg intramuscularly one-time depot shot was added, with a second dose five days later of 117 mg intramuscularly one time. She continued to endorse visual hallucinations of “the head of the devil” that “moves like a shadow”. Other notable findings were a Montreal Cognitive Assessment score of 22/30 with deficits in memory and executive functioning. Given the refractory nature of her disease and onset of new symptoms specifically of a visual nature, a head CT without contrast was ordered to rule out organic pathology. The images showed small bilateral basal ganglia calcifications, in addition to mild to moderate bilateral periventricular and deep white matter low attenuation suggestive for chronic small vessel ischemic disease (Figure 3).

On day 9 of admission, patient remained internally preoccupied with a disorganized thought process. Throughout the following week, she continued to hear voices of “the devil” with beliefs that the “evil spirits attack me and my husband”. Haloperidol 5 mg orally twice daily was added for treatment of psychosis, and no side effects of the medications were reported by the patient. Her antipsychotic regimen of fluphenazine 5 mg orally twice daily that was eventually increased to 7.5 mg orally twice daily, as delusions of persecution with auditory and visual hallucinations, continued to be present.

A differential diagnosis of psychosis due to a neurological condition was added, with possible role of basal ganglia injury considered in light of the visual hallucinations, akathisia symptoms, and cognitive dysfunction. The patient was treated symptomatically with fluphenazine Hcl (Prolixin) 5 mg orally twice daily, hydroxyzine Hcl 10 mg orally twice daily, and benztropine 1 mg twice daily. Although her visual hallucinations and delusions did not resolve significantly with medications, an augmentation with psychoeducation, supportive therapy, and cognitive behavior therapy helped the patient cope with her symptoms.

## 3. Discussion

The basal ganglia have been postulated to play a role in motor control, emotional processing, perception, and cognitive function [8]. Consequently, an insult to any part of this circuit should reveal abnormal symptoms of perception, emotional processing, and cognition. Our cases demonstrated progressive disturbances in mood and perception (particularly auditory and visual) in the context of basal ganglia pathology. The possibility of attributing these symptoms to basal ganglia pathology is informed by the evolving understanding of the role of the basal ganglia and brain imaging [8].

In the first and second cases, the late onset of the symptoms raises the index of suspicion for an underlying neurological cause. Mufaddel et al. reported that patients with BGC tend to develop psychiatric symptoms later in life than other psychiatric patients and have higher rates of medical comorbidities [9]. In the third case, the patient had a chronic diagnosis of schizophrenia. The presence of refractory symptoms and visual hallucinations usually motivate a medical work-up for underlying organic etiology which in her case revealed bilateral basal ganglia calcification like the other two cases. Cummings et al. [10] reported a case of basal ganglia calcification that presented with schizophrenia like symptoms as early as age of 17 years; similar observations have been noted in the literature [11]. The image findings reflected a pathology of the basal ganglia bilaterally along with peripheral calcifications of the posterior occipital lobe and central tentorium like those reported in prior literature [8, 10, 12, 13].

The similar natures of the three cases led the team to conduct a literature review on the varying symptoms reported in patients with basal ganglia pathology. Consequently, PubMed was searched for peer-reviewed articles that reported psychiatric symptoms and also had evidence of basal ganglia calcification(s). The search was not restricted by timeframe or language. The search was conducted using the keywords and MeSH terms: “basal ganglia” “calcification” and “function.” We also searched the reference list of eligible articles to identify additional papers relevant to this study. Screening for eligible articles was conducted independently by five authors. Eligible studies were those that focused on symptomatology of basal ganglia injury and respective behavior in human subjects.

Given the paucity of articles on this topic, all types of studies were considered for analysis including experimental, cohort, meta-analysis, case-control, case series, and case reports. Any disagreement regarding the eligibility of an article was resolved by discussion among the authors. Relevant data from eligible articles were extracted and entered onto a data abstraction form designed by the authors using Microsoft Excel. Data extracted from eligible articles include title of the article, gender and age of the patient, signs and symptoms elicited, and lab findings/imaging recorded. The literature review table is shown in Table 1.

Bilateral basal ganglia calcification was confirmed by CT scan in a total of 316 patients, ranging from 7 to 96 years old. 125 patients were male and 191 were female. Sixteen percent were ages 7-30 years old, 31% were ages between 20 and 50 years old, about 32% were ages 50 to 70 years old, and 21% were 70 years and older. In terms of location, 33% had no specification of the basal ganglia calcification, while 51% were in the globus pallidum and 18% in the putamen. Twenty-two percent of the cases had other areas of calcification outside the basal ganglia including the thalamus, cerebral white matter, the cerebellar dentate nuclei, and the putamen.

About 53% of cases reported no psychiatric symptoms. Of the 47% that reported symptoms, the symptoms included both affective symptoms (19% of cases) and psychotic symptoms (26% of cases). The most commonly reported symptoms included delusions (12%), extrapyramidal symptoms (9%), mood lability (9%), visual hallucinations (3%), and agitation (1%). Cognitive dysfunctions were reported in less than 1% of cases. Five percent of cases presented with symptoms that met the criteria for schizophrenia, and 2% presented with symptoms that met criteria for mania. No cross correlation was reported between the different symptoms. There was also no report of correlation between the size of the calcification, location of the calcifications in the basal ganglia, the duration of the calcifications, and the symptoms presented. In addition, there were no reports on the course and evolution of the symptoms reported in the literature. Further studies may be needed to explore these factors in understanding the neuropsychiatry of basal ganglia pathology.

*Limitations*. (1) To explore functional image studies that may also reveal dysfunction in other areas of the brain causing the pathology (2). Being a report of three individual cases, the need for a larger number of cases may provide a clearer picture of correlation.

## 4. Conclusion

A growing body of theoretical and clinical evidence shows that the basal ganglia play a key role in perceptual disturbances in addition to motor function. Our three cases demonstrate affective and psychotic symptoms concurrent with basal ganglia pathology. It is consistent with a literature review of the symptoms reported in patients with basal ganglia pathology. More appreciation of neuropsychiatric presentation of basal ganglia injury is needed with studies characterizing the correlation between basal ganglia pathology including onset and subnuclei location with symptoms. In doing so, we may improve the diagnosis, management and treatment following injury.

## Figures and Tables

**Table 1:** A review of selected literature on basal ganglia injury resulting in neuropsychiatric manifestations.

Researchers	Article Title	Number & Gender of Participants	Age	Signs & Symptoms	Lab Findings/Imaging Studies
Shirahama, M., Akiyoshi, J., Ishitobi, Y., et al.	A young woman with visual hallucinations, delusions of persecution and a history of performing arson with possible three-generation Fahr disease [14]	1 F	23	Visual hallucinations, delusion of injury, irritability, mood lability	CT: bilateral calcifications of the basal ganglia (globus pallidus)

Ber, I. L., Marié, R., Chabot, B., et al.	Neuropsychological and 18FDG-PET studies in a family with idiopathic basal ganglia calcifications [2]	5 M Pts. 3 F Pts.	7-73	Schizophrenia like psychosis with late EPS	CT & MRI: basal ganglia calcification

Pan, B., Liu, W., Chen, Q., et al.	Idiopathic basal ganglia calcification presenting as schizophrenia-like psychosis and obsessive-compulsive symptoms: A case report [15]	1 M	41	Schizophrenia-like psychosis and obsessive-compulsive symptoms	CT: symmetrical calcifications in the basal ganglia

Kasuga, K., Konno, T., Saito, K., et al.	A Japanese family with idiopathic basal ganglia calcification with novel SLC20A2 mutation presenting with late-onset hallucination and delusion [16]	1 F	73	Auditory Hallucinations, heard swear words of neighboring children, and delusions she has been eavesdropped on	CT: symmetric calcification in the basal ganglia, thalamus, white matter, occipital cortex, and cerebellar dentate nuclei

Chabot, B., Roulland, C., & Dollfus, S.	Schizophrenia and familial idiopathic basal ganglia calcification: a case report [12]	3 M 2 F	36 - 68 (36, 38, 39, 46, 68)	Schizophrenia relapse	CT: bilateral and symmetrical basal ganglia calcifications

Nicolas, G., Guillin, O., Borden, A., et al.	Psychosis revealing familial idiopathic basal ganglia calcification [17]	F	39	Auditory hallucinations and delusions	CT: basal ganglia calcifications in the proband and in her two asymptomatic parents.

Lauterbach, E. C., Spears, T., Prewett, M. J., et al.	Neuropsychiatric disorders, myoclonus, and dystonia in calcification of basal ganglia pathways [18]	2 M	Patient 1: 44 Patient 2: 40	Both patients had cognitive dysfunction, temporal lobe-like symptoms (including amnestic state, perceptual distortions, or complex visual hallucinations), and myoclonus	Patient 1: CT with contrast: Bilateral medial and lateral pallidal calcification anteromedially. Thyroid T3, I＇＇4, T, and thyroid-stimulating hormone were all normal. Patient 2: MRI: bilateral holo-anterior globus pallidus calcification measuring 1 cm on the right and 1/2 cm on the left at the time of examination.

Flint, J., & Goldstein, L. H.	Familial calcification of the basal ganglia: a case report and review of the literature [19]	Pt 1: M Pt 2: F	Patient 1: 31 Patient 2: 67	Delusions that celebrities were jealous of his song-writing talent and were threatening him, third person auditory hallucinations, thought broadcasting and the belief that people knew what he was thinking. Slight choreiform movements of the arms; repetitive movements were slow and poor coordination.	Serum calcium, phosphate, alkaline phosphatase and PTH were all normal. Radiologic evaluation: bilateral calcification of the globus pallidus.

Forstl, H., Krumm, B., Eden, S., et al.	Neurological disorders in 166 patients with basal ganglia calcification: a statistical evaluation [20]	59 M 107 F	16-86	No evidence of a significantly increased risk of dementia, cerebral infarction, epilepsy, vertigo, headache, or alcoholism	CTs: 97% of BGC group had calcification in the globus pallidus and 46% had calcification in the putamen. 25 BGC patients showed a circumscribed unilateral calcification of the globus pallidus, and 143 patients showed bilateral mineralization

Francis, A.	Familial Basal Ganglia Calcification and Schizophreniform Psychosis [21]	5 M 3 F	9-53 (9, 13, 27,32, 35,42,44,53)	Persecutory delusions, aggressive behavior and auditory and visual hallucinosis, coarse involuntary movements of the head and trunk, Parkinsonian facies and gait, flattened affect, elevation of mood, pressured speech, paranoia, EPS.	Skull X-ray: bilateral calcifications of the basal ganglia

Vakaet, A., Rubens, R., Reuck, J. D., et al.	Intracranial bilateral symmetrical calcification on CT-scanning [22]	1 F	57	Tonic fit, LOC, tongue biting, urinary incontinence (hx of epilepsy), global dementia, dysarthria, EPS (rigidity) in all four limbs, positive Chvostek's sign.	CT: extensive bilateral symmetrical calcification in the region of the basal ganglia, nuclei of the cerebellum and the cerebral and cerebellar white matter.

Geschwind, D. H., Loginov, M., & Stern, J. M.	Identification of a Locus on Chromosome 14q for Idiopathic Basal Ganglia Calcification (Fahr Disease) [23]	3 M 9 F	9-73 (9,10,38,40, 41,46,43,58, 52,50,70,76)	Psychosis and schizophreniform psychosis	Whole-genome scan using polymorphic microsatellite markers: Genomic study establishes the first chromosomal locus (IGBC1) and linkage analysis regarding age of onset of familial IBGC produced results consistent with genetic anticipation. CT scan: IBGC

Roiter, B., Pigato, G., & Perugi, G.	Late-onset Mania in a Patient with Movement Disorder and Basal Ganglia Calcifications: A Challenge for Diagnosis and Treatment [24]	1 M	58	Severe delirious-manic episode consisting of irritability, dysphoria, talkativeness, racing thoughts, hyperactivity, distractibility, grandiosity, persecutory delusions, psychomotor agitation, aggressiveness, insomnia, and mental confusion	CT: mild diffuse cortical atrophy and small basal ganglia calcifications but these findings were considered not clinically relevant. CT and brain MRI (for the second episode): moderate diffuse cortical atrophy, especially in frontal and occipital regions, posterior white matter lesions due to chronic vascular ischemia, and small bilateral pallidal calcifications

Johnson, J. M., Legesse, B., Camprodon, J. A., et al.	The clinical significance of bilateral basal ganglia calcification presenting with mania and delusions [25]	1 M	37	New-onset psychotic mania presented with increase in guilt, elated and expansive mood, pressured speech, grandiose delusions (i.e. claiming he was Christ), increased energy, erratic thoughts and activity, impulsive and risk-taking. Presence of delusions of reference.	Complete blood count, basic metabolic panel, liver function tests, TSH, CRP, ESR, were unremarkable, with negative RPR. Serum calcium and intact PTH were within normal limits. Urine drug screen was negative. He did have a positive ANA, at 1:30, in a nonspecific speckled pattern. A heavy-metal screen was negative for arsenic, lead, mercury, and cadmium. CT: small bilateral basal ganglia calcifications.

Philpot, M. P., & Lewis, S. W.	The Psychopathology of basal ganglia calcification [26]	12 M 24 F	Mean affected M- 60 Mean affected F- 71	No psychiatric diagnosis was specifically associated with BGC although calcification of the putamen and the caudate was only found in patients with functional disorders.	No abnormalities of calcium or phosphate metabolism were found.

Trautner, R. J., Cummings, J. L., Read, S. L., et al.	Idiopathic basal ganglia calcification and organic mood disorder [27]	5 M	Pt 1: 55 Pt 2: 47 Pt 3: 56 Pt 4: 56 Pt 5: 49	Organic mood changes, including one patient with secondary mania. Symptoms resemble those of other disorders affecting subcortical structures and support an association between mood, affect, cognition, and the extra-pyramidal nuclear system.	Lab studies were normal amongst all cases. CT of Case 1: dense basal ganglia calcification and periventricular gray matter structures. CT of Case 2: calcification of putamen in basal ganglia. CT of Case 3: calcification in the putamen, caudate, pulvinar, deep sulci of frontal cortex and dentate nuclei. Skull X-ray of Case 3: dense basal ganglia calcification. CT of Case 4: extensive basal ganglia calcification in caudate nuclei, thalami, and dentate nuclei of cerebellum. CT of Case 5: massive intracranial calcifications of basal ganglia, cerebellum, and areas of cerebral white matter.

Cummings, J. L., Gosenfeld, L. F., Houlihan, J. P., et al.	Neuropsychiatric disturbances associated with idiopathic calcification of the basal ganglia [10]	1 M	56	Schizophrenia like psychosis: auditory hallucinations, ideas of reference and influence, psychomotor retardation, and circumstantial speech. Depressed mood.	CT: calcifications symmetrically deposited in the putamen, the pulvinar, a few sulci of the frontal cortex and the dentate nuclei. Skull X-rays: dense calcifications of the basal ganglia and the dentate nuclei of the cerebellum. CSF protein was elevated

Shakibai, S. V., Johnson, J. P., & Bourgeois, J. A.	Fahr's Disease: An Incidental Finding in a Case Presenting with Psychosis [28]	1 M	24	Aggressive behavior, abusive language, smiling to self, talking to self and ghosts, fear of others, irregular sleep patterns, delusions of persecution, auditory hallucinations.	MRI: symmetrical large areas and foci of calcification in bilateral basal ganglia, thalami, cerebellar parenchyma and subcortical regions of bilateral cerebral hemispheres

König, P.	Psychopathological alterations in cases of symmetrical basal ganglia sclerosis [29]	25 M 37 F	Varies	31 cases initially presented neurological symptoms, 25 psychiatric, and 6 cases had no symptoms. Initial Symptoms in Bilateral Symmetrical Basal Ganglia Sclerosis (n = 62; 6 Cases Incidental Discovers) Primary Neurological 50%, Seizures 6%, TIA 6% syncopes 5% Paresis/apoplexia 10% Cephalea/vertigo 13%, EPS-movement disorders 10%, Primary Psychiatric 40%, Organic affective 21% Organic paranoid/ hallucinatory 1.6% Compulsions 1.6% Alcoholism 8%. Oligophrenia 1.6% Dementia 8%.	For both psychiatric and neurological symptoms, no correlations between either localization or volume of intracerebral calcification were noted, excepting cases of dementia, which showed larger hyperdensities. As basal ganglia calcification inflicts morphological damage to the CNS, a deterioration of brain function should occur and, in fact, was identified in terms of either neurological and/or psychiatric symptoms.

Shakibai, S. V., Johnson, J. P., & Bourgeois, J. A.	Paranoid Delusions and Cognitive Impairment Suggesting Fahr's Disease [7]	1 F	62	Multiple paranoid delusions; beliefs that people were controlling her by manipulating electrical dials, unknown other people were “listening through the walls” of her home, fears that her bed was “magnetized”.	CT: bilateral symmetric basal ganglia calcifications

“M”, males; “F”, females; “Pt”, patient; “MRI”, Magnetic Resonance Imaging; “CT”, computed tomography.

## References

[1] Middleton F. A., Strick P. L. (2000). Basal ganglia and cerebellar loops: motor and cognitive circuits. *Brain Research Reviews*.

[2] Le Ber I., Marié R.-M., Chabot B., Lalevée C., Defer G.-L. (2007). Neuropsychological and 18FDG-PET studies in a family with idiopathic basal ganglia calcifications. *Journal of the Neurological Sciences*.

[3] Kotz S. A., Schwartze M., Schmidt-Kassow M. (2009). Non-motor basal ganglia functions: A review and proposal for a model of sensory predictability in auditory language perception. *Cortex*.

[4] Adachi N., Watanabe T., Matsuda H., Onuma T. (2000). Hyperperfusion in the lateral temporal cortex, the striatum and the thalamus during complex visual hallucinations: single photon emission computed tomography findings in patients with charles bonnet syndrome. *Psychiatry and Clinical Neurosciences*.

[5] Middleton F. A., Strick P. L. (2000). Basal ganglia output and cognition: Evidence from anatomical, behavioral, and clinical studies. *Brain and Cognition*.

[6] Rosenblatt A., Leroi I. (2000). Neuropsychiatry of Huntington's disease and other basal ganglia disorders. *Psychosomatics*.

[7] Shakibai S. V., Johnson J. P., Bourgeois J. A. (2005). Paranoid delusions and cognitive impairment suggesting Fahr’s disease. *Psychosomatics*.

[8] Brown L. L., Schneider J. S., Lidsky T. I. (1997). Sensory and cognitive functions of the basal ganglia. *Current Opinion in Neurobiology*.

[9] Mufaddel A. A., Osman O. T., Al-Hassani G., Al-Bedwawi S., Hashim M. J. (2016). Basal ganglia calcification in psychiatric inpatients: a case-control study. *Cognitive and Behavioral Neurology*.

[10] Cummings J. L., Gosenfeld L. F., Houlihan J. P., McCaffrey T. (1983). Neuropsychiatric disturbances associated with idiopathic calcification of the basal ganglia. *Biological Psychiatry*.

[11] Caine E. D., Shoulson I. (1983). Psychiatric syndromes in Huntington's disease. *The American Journal of Psychiatry*.

[12] Chabot B., Roulland C., Dollfus S. (2001). Schizophrenia and familial idiopathic basal ganglia calcification: a case report. *Psychological Medicine*.

[13] Entezami P., Raff M., Bonner S. (2015). Visual hallucinations secondary to infarction of the caudate. *Journal of Neurology Neurophysiology*.

[14] Shirahama M., Akiyoshi J., Ishitobi Y. (2010). A young woman with visual hallucinations, delusions of persecution and a history of performing arson with possible three-generation Fahr disease. *Acta Psychiatrica Scandinavica*.

[15] Pan B., Liu W., Chen Q. (2015). Idiopathic basal ganglia calcification presenting as schizophrenia-like psychosis and obsessive-compulsive symptoms: a case report. *Experimental and Therapeutic Medicine*.

[16] Kasuga K., Konno T., Saito K., Ishihara A., Nishizawa M., Ikeuchi T. (2014). A Japanese family with idiopathic basal ganglia calcification with novel SLC20A2 mutation presenting with late-onset hallucination and delusion. *Journal of Neurology*.

[17] Nicolas G., Guillin O., Borden A., Bioux S., Lefaucheur R., Hannequin D. (2013). Psychosis revealing familial idiopathic basal ganglia calcification. *General Hospital Psychiatry*.

[18] Lauterbach E. C., Spears T. E., Prewett M. J., Price S. T., Jackson J. G., Kirsh A. D. (1994). Neuropsychiatric disorders, myoclonus, and dystonia in calcification of basal ganglia pathways. *Biological Psychiatry*.

[19] Flint J., Goldstein L. H. (1992). Familial calcification of the basal ganglia: A case report and review of the literature. *Psychological Medicine*.

[20] Förstl H., Krumm B., Eden S., Kohlmeyer K. (1992). Neurological disorders in 166 patients with basal ganglia calcification: a statistical evaluation. *Journal of Neurology*.

[21] Francis A. F. (1979). Familial basal ganglia calcification and schizophreniform psychosis. *The British Journal of Psychiatry*.

[22] Vakaet A., Rubens R., de Reuck J., vander Eecken H. (1985). Intracranial bilateral symmetrical calcification on CT-scanning. A case report and a review of the literature. *Clinical Neurology and Neurosurgery*.

[23] Geschwind D. H., Loginov M., Stern J. M. (1999). Identification of a locus on chromosome 14q for idiopathic basal ganglia calcification (Fahr disease). *American Journal of Human Genetics*.

[24] Roiter B., Pigato G., Perugi G. (2016). Late-Onset mania in a patient with movement disorder and basal ganglia calcifications: a challenge for diagnosis and treatment. *Case Reports in Psychiatry*.

[25] Johnson J. M., Legesse B., Camprodon J. A., Murray E., Price B. H. (2013). The clinical significance of bilateral basal ganglia calcification presenting with mania and delusions. *The Journal of Neuropsychiatry and Clinical Neurosciences*.

[26] Philpot M. P., Lewis S. W. (1989). The psychopathology of basal ganglia calcification. *Behavioural Neurology*.

[27] Trautner R. J., Cummings J. L., Read S. L., Benson D. F. (1988). Idiopathic basal ganglia calcification and organic mood disorder. *The American Journal of Psychiatry*.

[28] Srivastava S., Bhatia M. S., Sharma V., Mahajan S., Rajender G. (2010). Fahr's disease: An incidental finding in a case presenting with psychosis. *German Journal of Psychiatry*.

[29] König P. (1989). Psychopathological alterations in cases of symmetrical basal ganglia sclerosis. *Biological Psychiatry*.

